# Targeting Splicing in the Treatment of Human Disease

**DOI:** 10.3390/genes8030087

**Published:** 2017-02-24

**Authors:** Marc Suñé-Pou, Silvia Prieto-Sánchez, Sofía Boyero-Corral, Cristina Moreno-Castro, Younes El Yousfi, Josep Mª Suñé-Negre, Cristina Hernández-Munain, Carlos Suñé

**Affiliations:** 1 Department of Molecular Biology, Institute of Parasitology and Biomedicine “López Neyra” (IPBLN-CSIC), PTS, Granada 18016, Spain; marc@ipb.csic.es (M.S.-P.); silprie@ipb.csic.es (S.P.-S.); sofiaboyco@ipb.csic.es (S.B.-C.); cmcastro1988@ipb.csic.es (C.M.-C.); younes@ipb.csic.es (Y.E.Y.); 2 Drug Development Service, Department of Pharmacy and Pharmaceutical Technology, Faculty of Pharmacy, University of Barcelona, Avda. Joan XXIII, s/n 08028 Barcelona, Spain; jmsune@ub.edu; 3 Department of Cell Biology and Immunology, Institute of Parasitology and Biomedicine “López Neyra” (IPBLN-CSIC), PTS, Granada 18016, Spain; chmunain@ipb.csic.es

**Keywords:** alternative splicing, precursor messenger RNA, therapy, genetic disease

## Abstract

The tightly regulated process of precursor messenger RNA (pre-mRNA) alternative splicing (AS) is a key mechanism in the regulation of gene expression. Defects in this regulatory process affect cellular functions and are the cause of many human diseases. Recent advances in our understanding of splicing regulation have led to the development of new tools for manipulating splicing for therapeutic purposes. Several tools, including antisense oligonucleotides and *trans*-splicing, have been developed to target and alter splicing to correct misregulated gene expression or to modulate transcript isoform levels. At present, deregulated AS is recognized as an important area for therapeutic intervention. Here, we summarize the major hallmarks of the splicing process, the clinical implications that arise from alterations in this process, and the current tools that can be used to deliver, target, and correct deficiencies of this key pre-mRNA processing event.

## 1. Introduction

The Human Genome Project has revealed that there are approximately 20,000–25,000 protein-coding genes [1]. The Human Proteome Map project [2] has so far identified more than 30,000 proteins from approximately 293,000 peptides coded by approximately 17,000 human genes, and it is estimated that the diversity of these protein-coding genes in humans is greatly increased by the presence of numerous protein variants (isoforms). Several mechanisms are responsible for the formation of protein isoforms, such as genetic variations, somatic recombination, post-translational and proteolytic modifications, and alternative splicing (AS).

Genes are composed of introns and exons, but only exons contain the information necessary to make proteins. AS of the pre-messenger RNA (mRNA) is a process by which the introns are removed and the exons are appropriately aligned and ligated to form the mRNA. Human genes are composed of an average of 7–8 introns and 8–9 exons. Considering that 3000 genes are actively transcribed at any given moment and that every gene is capable of producing at least three transcripts, this results in more than 60,000 introns that need to be simultaneously spliced [3]. Therefore, this process needs to be efficiently spatiotemporally coordinated to yield a mature mRNA that is exported from the nucleus to the cytoplasm to be translated into protein.

In humans, >90% of genes undergo AS [4,5], underscoring the fundamental importance of this regulatory process in expanding protein diversity through evolution. As such, the misregulation of AS by mutations that affect the splicing signals or the splicing machinery itself is the cause of multiple human diseases [6,7,8]. In this review, we aim to revisit current concepts and experimental observations in therapeutic strategies to treat diseases arising from splicing defects, including advances in the emerging field of nanotechnology.

## 2. Pre-mRNA Splicing

**Figure 1:**
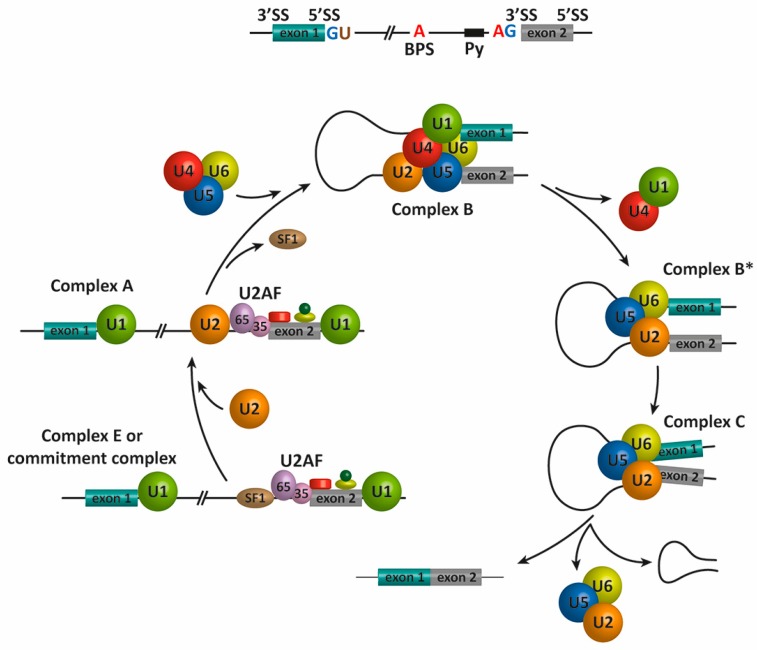
Schematic representation of the spliceosome assembly and pre-mRNA splicing. In the first step of the splicing process, the 5′ splice site (GU, 5′ SS) is bound by the U1 snRNP, and the splicing factors SF1/BBP and U2AF cooperatively recognize the branch point sequence (BPS), the polypyrimidine (Py) tract, and the 3′ splice site (AG, 3′ SS) to assemble complex E [11,12]. The binding of the U2 snRNP to the BPS results in the pre-spliceosomal complex A [13]. Subsequent steps lead to the binding of the U4/U5–U6 tri-snRNP and the formation of complex B [14]. Complex C is assembled after rearrangements that detach the U1 and U4 snRNPs [15] to generate complex B*. Complex C is responsible for the two trans-esterification reactions at the SS. Additional rearrangements result in the excision of the intron, which is removed as a lariat RNA, and ligation of the exons. The U2, U5, and U6 snRNPs are then released from the complex and recycled for subsequent rounds of splicing [16,17].

The splicing reaction is catalyzed by the spliceosome, a macromolecular complex formed by five small nuclear ribonucleoproteins (snRNPs), termed U1, U2, U4, U5, and U6, and approximately 200 proteins [9]. The assembly of the spliceosome on pre-mRNA includes the binding of U1 snRNP, U2 snRNP, the pre-formed U4/U6-U5 triple snRNP, and the Prp19 complex [10]. This assembly occurs through the recognition of several sequence elements on the pre-mRNA that define the exon/intron boundaries, which include the 5′ and 3′ splice sites (SS), the associated 3′ sequences for intron excision, the polypyrimidine (Py) tract, and the branch point sequence (BPS). The assembly of the spliceosome during the process of AS is depicted in Figure 1. In mammals, the first catalytic step of the splicing reaction begins when the U1 snRNP binds the 5′ SS of the intron (defined by the consensus sequence AGGURAGU), and the splicing factors SF1 and U2AF cooperatively recognize the BPS, Py, and 3′ SS to assembled complex E or the commitment complex [11,12]. Subsequently, U2 snRNP and additional proteins are recruited to the pre-mRNA BPS to form the pre-spliceosome or complex A [13]. The binding of the U4/U6-U5 tri-snRNP forms the pre-catalytic spliceosome or complex B [14]. After RNA-RNA and RNA-protein rearrangements at the heart of the spliceosome, U1 and U4 are released [15] to form the activated complex B or complex B* This complex is responsible for executing the first catalytic step, through which the phosphodiester bond at the 5′ SS of the intron is modified by the 2′-hydroxyl of an adenosine of the BPS to form a free 5′ exon and a branched intron, which is subsequently degraded. During this process, additional rearrangements occur to generate the catalytic spliceosome or complex C, which is ultimately responsible for catalyzing the intron excision and exon–exon ligation reactions. After the second catalytic step, the U2, U5, and U6 snRNPs are released from the post-spliceosomal complex and recycled for additional rounds of splicing [16,17].

**Figure 2:**
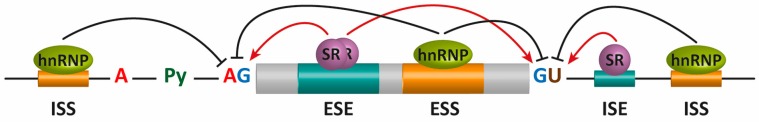
AS regulation by *cis* elements and *trans*-acting factors. The core *cis* sequence elements that define the exon/intron boundaries (5′ and 3′ splice sites (SS), GU-AG, polypyrimidine (Py) tract, and branch point sequence (BPS)) are poorly conserved. Additional enhancer and silencer elements in exons and in introns (ESE: exonic splicing enhancers; ESI: exonic splicing silencers; ISE: intronic splicing enhancers; ISI: intronic splicing silencers) contribute to the specificity of AS regulation. *Trans*-acting splicing factors, such as SR family proteins and heterogeneous nuclear ribonucleoprotein particles (hnRNPs), bind to enhancers and silencers and interact with spliceosomal components [18,19,20]. In general, SR proteins bound to enhancers facilitate exon definition, and hnRNPs inhibit this process.

We know that additional sequence elements, known as exonic and intronic splicing silencers or enhancers (ESS, ISS, ESE, and ISE, respectively), participate in the regulation of AS. Specific RNA-binding proteins, including heterogeneous nuclear ribonucleoproteins (hnRNPs) and serine/arginine-rich (SR) proteins, recognize these sequences to positively or negatively regulate AS (Figure 2). These regulators, together with an ever-increasing number of additional auxiliary factors, provide the basis for the specificity of this pre-mRNA processing event [18,19,20].

**Figure 3:**
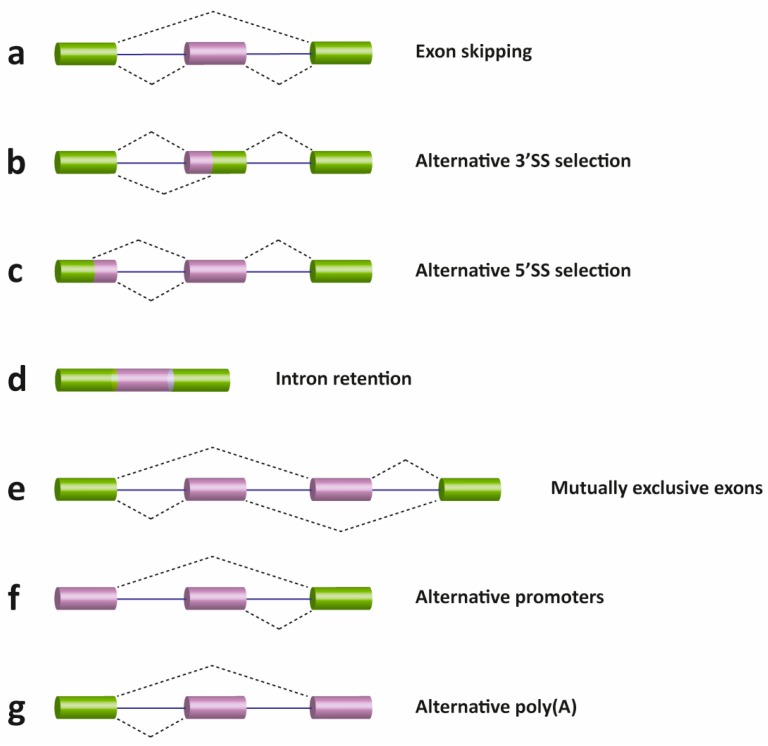
Schematic representation of different types of alternative transcriptional or splicing events, with exons (boxes) and introns (lines). Constitutive exons are shown in green and alternatively spliced exons in purple. Dashed lines indicate the AS event. Exon skipping (**a**); alternative 3′ (**b**) and 5′ SS selection (**c**); intron retention (**d**); mutually exclusive exons (**e**); alternative promoter usage (**f**); and alternative polyadenylation (**g**) events are shown. Like alternative splicing (AS), usage of alternative promoter and polyadenylation sites allow a single gene to encode multiple mRNA transcripts.

There are several different types of AS events, which can be classified into four main subgroups. The first type is exon skipping, which is the major AS event in higher eukaryotes. In this type of event, a cassette exon is removed from the pre-mRNA (Figure 3, panel a). The second and third types are alternative 3′ and 5′ SS selection (Figure 3, panel b and c). These types of AS events occur when the spliceosome recognizes two or more splice sites at one end of an exon. The fourth type is intron retention (Figure 3, panel d), in which an intron remains in the mature mRNA transcript. This AS event is much more common in plants, fungi and protozoa than in vertebrates. Other events that affect the transcript isoform outcome include mutually exclusive exons (Figure 3, panel e), alternative promoter usage (Figure 3, panel f), and alternative polyadenylation (Figure 3, panel g).

## 3. Connections between Splicing and Human Disease

As stated above, several diseases are caused by mistakes in the splicing process. These diseases can be classified in two types depending on their origin: mutations in *cis* elements (i.e., affecting the splicing signals) and mutations in *trans* elements (i.e., affecting the splicing machinery itself).

Mutations in the core splicing consensus sequences are known to lead to diseases. These mutations can produce changes in the 5′ and 3′ SS and surrounding sequences or in the BPS or generate new SS that may lead to disease when they are used. Alterations in the auxiliary *cis* elements described above (the exonic and intronic splicing silencers or enhancers) may also lead to aberrant AS and cause disease.

As described above, the splicing reaction is a highly orchestrated process that requires the fine-tuned coordination of a great number of proteins. Mutations in core spliceosome components or auxiliary factors may disrupt, this mechanism in the cellular regulatory network and lead to diseases. Because several reviews covering dysfunctions related to AS have been published elsewhere [7,8,21,22], we do not aim to discuss specific examples here.

## 4. Therapeutic Approaches

Gene therapy has emerged as a promising pharmacotherapy option for patients with diseases of genetic origin. During the last several decades, a diverse array of approaches to genetically modifying a cell or organism has been investigated. Next, we discuss some strategies to treat diseases that have been used to modify and fix errors in the splicing process and provide a summary of some diseases affecting this process that might be a target for gene therapy (Table 1).

### 4.1. Antisense Oligonucleotides (ASOs)

**Figure 4:**
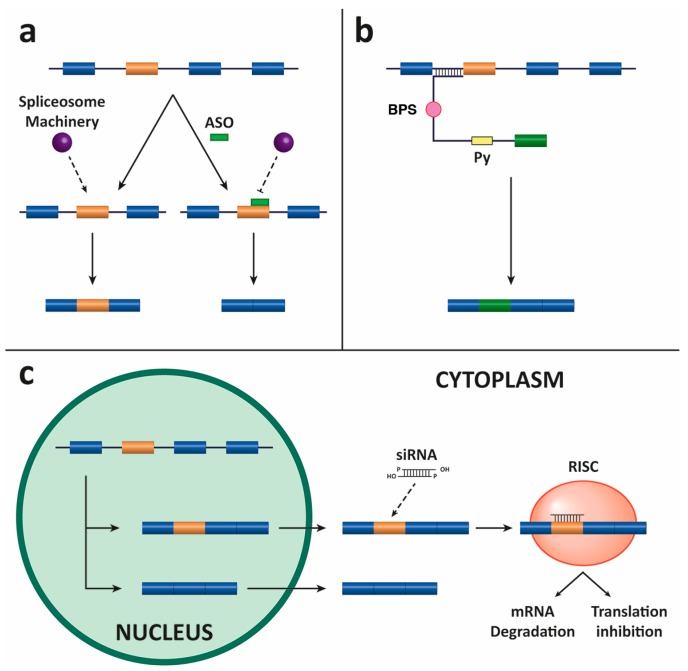
Three different strategies to target splicing for gene modification. (**a**) The diagram depicts an antisense oligonucleotide (ASO)-based strategy to target an alternatively spliced exon (in orange). In the absence of the ASO, the spliceosome is assembled and the exon is included in the mRNA; in the presence of the ASO, the spliceosome is sterically blocked and the exon is skipped and not included in the mRNA. (**b**) SMaRT strategy for *trans*-splicing by 5′ exon replacement. Schematic representation of the gene-specific pre-*trans*-splicing molecule (PTM). The coding sequence of the PTM consists of an exon (in green), and the *trans*-splicing domain of the PTM comprises a binding-domain (BD) complementary to the 3′ end of the gene intron as well as highly conserved BPS and Py sequences. (**c**) Illustration depicting the mechanism by which siRNA can inhibit the expression of specific exon-containing target gene products by hybridizing to the mRNA and triggering RISC-mediated degradation or translational inhibition.

This strategy is based on short oligonucleotides that are guided to the pre-mRNA to modify the splicing process. ASOs can be designed to target the SS or auxiliary sequences (see above) to modify the outcome of the splicing reaction, thereby leading to mRNA repair and the restoration of protein function [36]. These splicing-related ASOs act through a different mechanism of action than conventional antisense oligonucleotides or siRNA (see below), which inhibit gene expression by degrading the target mRNA. These sequences sterically block relevant motifs in the pre-mRNA without promoting degradation while shifting the splicing outcome (Figure 4, panel a). For this reason, they are also called splice-switching oligonucleotides (SSOs).

Another splice switching strategy is to fuse the RNA sequence that is complementary to an RNA binding motif (the ASO) with an untethered RNA segment that serves as a sequence-specific binding platform for the recruitment of a splicing silencer or activator to the targeted RNA (TOSS and TOES, respectively, for targeted oligonucleotide silencer of splicing and targeted oligonucleotide enhancer of splicing) [37]. This approach was used to redirect the splicing to favor the inclusion of endogenous exon 7 SMN2 transcript to increase the level of functional SMN protein [38] and to alter the AS of the BCL2L1 pre-mRNA to promote apoptosis in cancer cells in culture [39]. These bifunctional oligonucleotides to alter splicing decisions can be used against a wide range of targets [40]. Frequently, ASOs are chemically modified to improve binding affinity and avoid degradation.

Among the best characterized medical examples of the use of ASOs is in the treatment of Duchenne muscular dystrophy (DMD). DMD is a genetic disorder characterized by progressive muscle degeneration and weakness caused by the alteration of dystrophin, which anchors the extracellular matrix to the cytoskeleton of muscle fibers. The *DMD* gene is the largest known human gene with 79 exons. Deletion mutations have been identified in approximately two-thirds of DMD cases and the clinical variation in phenotype correlates with the maintenance or disruption of the translational open reading frame of the mRNA [41]. An antisense-mediated approach to restore the reading frame by targeting exons flanking frame-shift deletions functionality was one of the methods devised early on [42,43]. These early promising results were confirmed by several other studies using antisense-mediated restoration of the reading frame as a therapy for Duchenne patients [44,45,46]. Very recently, a phosphorodiamidate morpholino oligomer (PMO) designed to induce exon 51 skipping (Eteplirsen/Exondys 51) has received accelerated approval from the U.S. Food and Drug Administration (FDA) for the treatment of DMD [47,48].

Spinal muscular atrophy (SMA) is a disease caused by mutations and deletions in the survival motor neuron 1 (*SMN1*) gene that can be partially compensated for by increasing the inclusion of exon 7 in the second copy gene *SMN2*. In a series of seminal articles, Krainer’s group showed that appropriate 2′-*O*-(2-methoxyethyl) (MOE) phosphorothioate-modified ASOs can efficiently correct *SMN2* exon 7 splicing. Importantly, this splicing correction was achieved in cultured human cells (including patient fibroblasts) and in induced mouse models of SMA [49,50,51,52]. These results led to the development of nusinersen (Spinraza), an ASO that after extensive preclinical and clinical testing [53] has been approved on December 23 by the FDA under Priority Review for the treatment of SMA in pediatric and adult patients. These are perfect examples of how basic research focused on the mechanisms of disease is key to important clinical developments.

Antisense derivatives of U7 snRNP can also redirect splicing towards the synthesis of the exon 7-containing SMN2 protein in cultured cells and SMA mouse models [54,55], which support the usefulness of ASOs as promising therapeutic drugs [56].

The ASO strategy has also been successfully used for targeting the pre-mRNA to restore prematurely stopped open reading frames, such as in the inherited disease dystrophic epidermolysis bullosa (DEB) [57], induce isotype switching of the Tau mRNA in the frontotemporal dementia and parkinsonism linked to chromosome 17 (FTDP-17) [58], generate a truncated APOB100 protein with therapeutic utility to prevent the development of atherosclerosis [59], or induce exon inclusion of the exon necessary for the treatment of cystic fibrosis [60].

### 4.2. Spliceosome-Mediated RNA Trans-Splicing (SMaRT)

SMaRT is a system used to reprogram mRNA that introduces into cells the part of the mutant transcript that has to be corrected instead of a full-length cDNA sequence. In brief, the SMaRT technology needs three components. Two of them are provided by the cell: the spliceosome machinery and the target mRNA. The third component of the system, which has to be introduced into the cell, is the pre-*trans*-splicing molecule (PTM) (also termed RNA *trans*-splicing molecule or RTM). The final goal is to recombine the endogenous mutated target pre-mRNA with the exogenous PTM and achieve the substitution of the mutated region for the wild-type sequence [61]. Designing a correct PTM is crucial for the reprogramming of mRNA. The PTM must carry the wild-type coding region of the gene that is to be replaced, 5′ and 3′ SS, intronic BPS and Py sequences, and a complementary sequence or binding domain for precise and specific hybridization to the mutated pre-mRNA (Figure 4, panel b) [62,63].

There are three types of SMaRT approaches: 5′-*trans*-splicing, 3′-*trans*-splicing, and internal exon replacement (IER), which target the 5′-, 3′-, or internal portion of a mutated target pre-mRNA, respectively. In the last few years, there have been several examples of the use of the SMaRT strategy as a tool for treating genetic diseases. Early pioneering work using HeLa nuclear extracts, cultured human lung cancer cells, and tumor-bearing athymic (nude) mice suggested that SMaRT could represent a general approach for reprogramming the sequence of targeted transcripts [64]. Subsequently, the same group demonstrated the feasibility of the system by repairing mutations in the cystic fibrosis *trans*-membrane conductance regulator (CFTR) gene using a 5′-exon replacement approach [65], which was followed by a study showing that SMaRT can efficiently promote the production of a functional protein in vitro [66]. Another example of using SMaRT to reprogram mRNA is the correction of the Tau isoform imbalance that is characteristic of FTDP-17 and tauopathies [67,68,69]. Recently, the *trans*-splicing system has been optimized through the combination of the trans-splicing RNA and antisense RNA interfering with competitive splicing elements on the pre-mRNA [70,71]. A comprehensive review detailing the use of SMaRT in gene therapy for genetic diseases has been recently published [63].

### 4.3. Small Interfering RNAs (siRNAs)

One of the most important advances in the field of molecular biology is the use of siRNAs to silence the expression of genes. The administration of RNA of 21–23 nucleotides (nt) in length can prevent the translation of an endogenous mRNA through its base pairing with the target to induce degradation or translation inhibition depending on the degree of complementarity (Figure 4, panel c). Targeting aberrant splicing isoforms is one of the many potential therapeutic uses of siRNAs. Exonic, intronic, and exonic/intronic junction sequences have been used to design siRNAs to specifically degrade aberrant or alternatively spliced mRNAs. This targeting approach was used, among others, in the fibronectin gene, Ullrich congenital muscular dystrophy (UCMD), and growth hormone deficiency (GHD) type II diseases [72,73,74].

Some of the mentioned examples about therapeutic approaches to modulate splicing are summarized in Table 2.

## 5. Delivery Methods

One of the main challenges in gene therapy is the delivery of foreign genes to human patients. Today, viral and non-viral methods are used for delivering genes and biomolecules in vivo. Both approaches have advantages and disadvantages, which we briefly analyze.

### 5.1. Viral Methods

The use of viruses such as retrovirus, adenovirus or adeno-associated virus (AAV), among others, is one of the most successful gene therapy systems available today. The administration of viral vectors to human patients is done by direct injection into target tissues or by the injection of viral-modified cells [75]. The most important advantage of viruses as vectors for gene delivery is their high transfection efficiency. However, the system has some disadvantages, such as their marked immunogenicity, the phenotoxicity of the transgene, and potential vertical and horizontal transmission by replication-competent viruses. More recently, viruses have been appropriately modified to minimize the associated immunogenicity while introducing the therapeutic gene unit in its genome, e.g., by replacing viral pathogenic elements [76]. When using retroviral and lentiviral vectors, integration into the host genome poses a risk of vector-mediated alterations in cells that are relevant for gene therapy applications. In support of this statement, a whole transcriptome analysis of aberrant splicing events occurring upon lentiviral vector transduction has been reported [77]. Multi-drug resistance elicited by viral vectors, such as Adenovirus, may affect the efficacy of chemotherapy, thus limiting the use of these vectors [78]. Problems with scaling up production processes and high economic costs are also barriers that need to be improved when considering viral vectors to treat diseases. Several recent reviews covering specific aspects of the use of viral vectors for gene therapy have been published elsewhere [75,79,80]. Aspects such as the insert size, time-course of transgene expression, route administration, and gene targeting are of primary importance when choosing the right viral vector.

Viral vectors have been used in certain cases of human diseases by changing the splicing pattern (even if the origin of the disease is not strictly related to the splicing process) such in SMA [81,82,83], DMD [84,85,86,87,88], cystic fibrosis [89,90], retinitis pigmentosa [91,92], aromatic L-amino acid decarboxylase (AADC) deficiency [93], UDP *N*-acetylglucosamine 2-epimerase/*N*-acetylmannosamine kinase (*GNE*) myopathy [94], Fanconi anemia C (FANCC) [95], and retinal disease [96].

### 5.2. Non-Viral Methods

In recent decades, nanoparticle-mediated delivery of biomolecules has received much attention for its potential to modulate the regulation of gene expression for the treatment of diseases, thus representing a promising new avenue for gene therapy [97,98,99,100]. The advantages of non-viral gene delivery systems compared to viral systems are clear in terms of the immunogenic responses. Whereas low immunogenicity is a positive trait, one obstacle of these non-viral delivery systems is the low transfection efficiency [101]. There are few reports where nanoparticles have achieved high gene transfection efficiency with values approaching those obtained using viral vectors [102].

**Figure 5:**
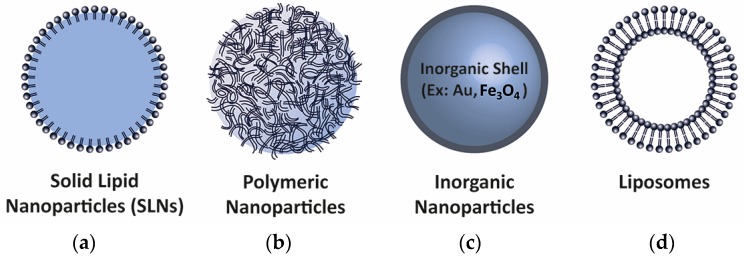
Schematic representation of different types of nanoparticles used to deliver biomolecules. (**a**) Solid lipid nanoparticles (SLNs); (**b**) Polymeric nanoparticles; (**c**) Inorganic core-shell nanoparticles (Au: gold, Fe_3_O_4_: iron oxide); (**d**) Lipid bilayer-based liposomes.

In recent years, the use of nanodelivery systems of different materials and the physiochemical properties necessary for the cellular uptake of biomolecules has become particularly important. There are a variety of different nanoparticle types, depending on manufacturing processes and components (Figure 5) [103]. The nanoparticle formula can be designed to produce carriers for oral [104], skin [105], liver [106], pulmonary [107], brain [108], or cancer targeting [109]. Nanoparticles can be used to deliver a combination of biomolecules to enhance the therapeutic effect against disease. For example, Liu et al. developed multifunctional nanoparticles carrying an inhibitory peptide and an shRNA for the treatment of Alzheimer’s disease, thus acting in relevant pathways in the pathogenesis of the disease [110]. The successful co-delivery of biomolecules overcoming the blood–brain barrier makes this type of nanostructured system useful for improving therapeutic delivery to the brain [108].

Nanoparticles can be used for delivering RNA in order to achieve controlled and selective therapeutic effects acting on the splicing process. To deliver these nanoparticles into cells where they display full bioactivity at nontoxic concentrations, recent studies have focused on understanding the effects of nanoparticle physicochemical properties [111]. Several formulations have been shown to effectively target diseased tissues to redirect the AS of pre-mRNA. Administration of lipid nanoparticles with *BCL2L1* ASO resulted in modification of *BCL2L1* pre-mRNA splicing to induce apoptosis and subsequent cell death in lung metastases. Redirection of *BCL2L1* pre-mRNA splicing was associated with reduced tumor load [112]. Chitosan-based nanoparticles have been successfully used to deliver intronic ASOs into embryonic and lymphoblastoid cells to modulate ataxia-telangiectasia mutated (*ATM*) gene expression [113], which is an interesting and promising target for anticancer therapy. In mice, cationic polymethylmethacrylate (PMMA) nanoparticles loaded with 2′*O*MePS delivered by intraperitoneal injections could restore dystrophin expression in skeletal and cardiac muscle [114,115], thereby showing the potential of this method for ASO delivery in DMD. Functional delivery by polyethylenimine nanoparticles of ASO conjugated to a bivalent arginine–glycine-aspartic acid (RGD) peptide, which specifically binds to integrin αvβ3, demonstrated dramatic increase in the pharmacological response of splicing correction through integrin-mediated endocytosis and rapid endosomal release [116]. Polyamidoamine (PAMAM) dendrimers are cationic polymers that have also been used to deliver 2′-*O*-methyl antisense oligonucleotides to correct splicing at an aberrant intron inserted into a luciferase reporter gene [117,118]. In conclusion, these results show great promise of using nanomaterials as nucleic acid vehicles to target and interfere with the splicing process.

Despite these remarkable in vitro and in vivo results using nanoparticle-mediated delivery to target splicing events, the translational progress for medical applications has been limited. Poor delivery efficiency and the inability to control the nanoparticle transport inside the body are major limitations that need to be overcome for the clinical translation of nanomedicine [119].

## 6. Conclusions

AS is an essential component of gene expression regulation that contributes to the diversity of cell and tissue-specific protein expression profiles. In recent decades, we have increased our knowledge of the mechanisms and compositional dynamics of how exons are alternatively spliced to generate a plethora of transcript isoforms. These studies give rise to important new basic questions regarding AS regulation to better understand this interesting and critical aspect of RNA biogenesis. Because of the connections between AS and disease, further studies are necessary to provide relevant insights into the molecular mechanisms involved in human disease. A step of considerable importance and an exciting concept is the possibility of targeting the splicing process for therapy. As outlined in this review, several approaches have been devised to modify the outcome of the splicing reaction to treat genetic diseases caused by splicing errors. Extensive studies have been performed, and a series of benchmark results have shown the proof of concept and the feasibility of this approach to make a real impact on gene therapy. However, these laboratory studies will unlikely translate to the clinic without a specific, efficient, and safe delivery system easily translatable to human patients. In recent years, the use of nanoparticles as transport systems for the delivery of drugs and biomolecules has received much attention, especially to detect and destroy cancer cells. Although nanotechnology has been successfully used for the delivery of molecules to redirect the splicing of pre-mRNA, it is important to address the barriers associated with delivery efficiency and transport inside the body to accelerate the clinical translation of this innovative splicing-targeting approach for therapy.

## Figures and Tables

**Table 1:** Summary of splicing-related diseases that might be target for gene therapy.

Disease		Regulatory Element Mutated	Mechanism	Splicing Effect	References
Familial dysautonomia (FD)		*Cis*	T > C mutation at position 6 of intron 20 of the *IKBKAP* gene	Exon skipping; introduction of a premature termination codon (PTC)	[23]
Spinal muscular atrophy (SMA)		*Cis*	C > T mutation at position 6 of exon 7 of the *SMN2* gene	Alteration of a putative ESE	[24]
Medium-chain acyl-CoA dehydrogenase (MCAD) deficiency		*Cis*	c362C > T mutation in exon 5 of the *MCAD* gene	Exon skipping	[25]
Hutchinson-Gilford progeria syndrome (HGPS)		*Cis*	c1824C > T mutation in exon 11 of *LMNA* gene	Activation of a cryptic splice site	[26]
Myotonic dystrophy	Type 1 (DM1)	*Cis*	Expanded CTG tract in the 3′ UTR region of the *DMPK* gene	Misregulation of *trans-*acting factors	[27]
	Type 2 (DM2)	*Cis*	Expanded CCCTG tract in intron 1 of the *ZNF9* gene	Misregulation of *trans*-acting factors	[27]
Autosomal dominant retinitis pigmentosa (RP)		*Trans*	Mutations in genes of the core spliceosome (*PRPF31*, *PRPF8*, *PRPF3*, *RP9*)	Disruption of basal spliceosome function	[28]
Duchenne muscular dystrophy (DMD)		*Cis*	T > A mutation in exon 31 of the Distrophin gene	Creation of a PTC and introduction of ESS	[29]
Microcephalic steodysplastic primordial dwarfism type 1 (MOPD1) or Taybi-Linder syndrome (TALS)		*Trans*	Mutations in the gene encoding the U4atac snRNA	Reduced splicing efficiency and increased intron retention	[30]
Frontotemporal dementia with parkinsonism-17 (FTDP-17)		*Cis*	Mutations within and downstream exon 10 of the *MAPT* gene	Disruption of Tau protein balance	[31]
Fukuyama congenital muscular dystrophy (FCMD)		*Cis*	SVA insertion in the 3′ UTR of the *FKTN* gene	Inclusion of a new exon	[32]
Amyotrophic lateral sclerosis (ALS)		*Trans*	Mutations in TDP-43	Altered gene splicing	[33]
Hypercholesterolemia		*Cis*	rs688T > C mutation in exon 12 of the *LDLR* gene	Alteration of ESE and exon skipping	[34]
Cystic fibrosis (CF)		*Cis*	Longer (UG)n tract at the exon 9 3′ SS of the CFTR gene	Exon skipping	[35]

**Table 2:** Examples of splicing-based therapeutic approaches (see text for details).

Disease	Therapeutic Approach	Target Gene	Regulated Exon
DMD	ASO	*DMD*	51
SMA	ASO	*SMN2*	7
Dystrophic epidermolysis bullosa (DEB)	ASO	*COL7A1*	70
FTDP-17	ASO	*MAPT*	10
	SMaRT	*MAPT*	1
Atherosclerosis	ASO	*APOB*	27
CF	ASO	*CFTR*	16
	SMaRT	*CFTR*	10
Ullrich congenital muscular dystrophy (UCMD)	siRNA	*COL6A3*	16
Growth hormone deficiency (GHD) type II	siRNA	*GH1*	3

## Abbreviations

The following abbreviations are used in this manuscript:

AS	alternative splicing
pre-mRNA	precursor messenger RNA
mRNA	messenger RNA
snRNPs	small nuclear ribonucleoproteins
SS	splice sites
Py	Polypyrimidine
BPS	branch point sequence
ISS, ISE, ESS, and ESE	exonic and intronic splicing silencers or enhancers
ASO	antisense RNA
SMaRT	spliceosome-mediated RNA *trans*-splicing
PTM	pre-*trans*-splicing molecule
siRNAs	small interfering RNAs
